# Synthesis and Characterisation of Hydrogels Based on Poly (N-Vinylcaprolactam) with Diethylene Glycol Diacrylate

**DOI:** 10.3390/gels9060439

**Published:** 2023-05-25

**Authors:** Elaine Halligan, Billy Shu Hieng Tie, Declan Mary Colbert, Mohamad Alsaadi, Shuo Zhuo, Gavin Keane, Luke M. Geever

**Affiliations:** 1 Polymer, Recycling, Industrial, Sustainability and Manufacturing (PRISM) Center, Technological University of the Shannon, Midlands Midwest, Dublin Road, N37 HD68 Athlone, Co. Westmeath, Ireland; 2 CONFIRM Centre for Smart Manufacturing, University of Limerick, V94 T9PX Limerick, Co. Limerick, Ireland; 3 Centre for Industrial Service and Design, Technological University of the Shannon, Midlands Midwest, Dublin Road, N37 HD68 Athlone, Co. Westmeath, Ireland; 4 Applied Polymer Technologies Gateway, Material Research Institute, Technological University of the Shannon, Midlands Midwest, Dublin Road, N37 HD68 Athlone, Co. Westmeath, Ireland

**Keywords:** N-vinylcaprolactam, photopolymerisation, hydrogel, copolymers, swelling behaviour

## Abstract

Poly (N-vinylcaprolactam) is a polymer that is biocompatible, water-soluble, thermally sensitive, non-toxic, and nonionic. In this study, the preparation of hydrogels based on Poly (N-vinylcaprolactam) with diethylene glycol diacrylate is presented. The N-Vinylcaprolactam-based hydrogels are synthesised by using a photopolymerisation technique using diethylene glycol diacrylate as a crosslinking agent, and Diphenyl (2, 4, 6-trimethylbenzoyl) phosphine oxide as a photoinitiator. The structure of the polymers is investigated via Attenuated Total Reflectance–Fourier Transform Infrared Spectroscopy. The polymers are further characterised using differential scanning calorimetry and swelling analysis. This study is conducted to determine the characteristics of P (N-vinylcaprolactam) with diethylene glycol diacrylate, including the addition of Vinylacetate or N-Vinylpyrrolidone, and to examine the effects on the phase transition. Although various methods of free-radical polymerisation have synthesised the homopolymer, this is the first study to report the synthesis of Poly (N-vinylcaprolactam) with diethylene glycol diacrylate by using free-radical photopolymerisation, using Diphenyl (2, 4, 6-trimethylbenzoyl) phosphine oxide to initiate the reaction. FTIR analysis shows that the NVCL-based copolymers are successfully polymerised through UV photopolymerisation. DSC analysis indicates that increasing the concentration of crosslinker results in a decrease in the glass transition temperature. Swelling analysis displays that the lower the concentration of crosslinker present in the hydrogel, the quicker the hydrogels reach their maximum swelling ratio.

## 1. Introduction

Hydrogels are highly hydrophilic polymer crosslinking networks that have high water content and good biocompatibility. Consisting of three-dimensional polymeric networks, hydrogels possess the ability to swell in water and hold large amounts of water while maintaining structural integrity, owing to their hydrophilic groups, such as –NH_2_, COOH, –OH, –CONH_2_, –CONH, and –SO_3_H [1]. The preparation of hydrogels can be achieved by physical or chemical crosslinking. Hydrogels that are physically crosslinked undergo a transition from a liquid to a gel state when the environmental conditions change, such as temperature, pH, ionic concentration, or a combination of two components. Chemically crosslinked hydrogels use covalent bonding, which initiates mechanical integrity and degradation resistance. The formation of hydrogels is understood to be due to the interactions between hydrophilic molecules to form a network that entraps water molecules. For a material to be considered a hydrogel, water must make up at least 10% of the total weight (or volume) of the material [2]. The literature reports several classifications of hydrogels: One classification can be based on the structure of the hydrogel in terms of amorphous, semi-crystalline, crystalline, and hydrocolloid aggregates [3]. Further classification can be performed based on their response mechanism: physically, chemically, and biochemically responsive hydrogels. The flexibility of hydrogels makes them very advantageous across many fields, especially in drug delivery, tissue engineering, actuators, wound dressing, contact lenses, and biosensors [4].

One interesting route of hydrogel synthesis is photopolymerisation. Due to the chemical reactions being insensitive to water, this method allows for work to be performed in aqueous media. The rapid processing time of hydrogel synthesis via photopolymerisation enables the synthesis to be completed within minutes. Additionally, hydrogel synthesis via photopolymerisation enables the regulation of spatial and temporal control over the crosslinking properties of the hydrogel [5]. Achieving photopolymerisation of hydrogels using UV radiation is one of the most widely used techniques today. This is due to the technique’s ease of operation, cost-effectiveness, and environmentally friendly nature [6]. In order to successfully photopolymerise hydrogels via UV radiation, the addition of a photoinitiator is required. Upon exposure to UV light, the photoinitiator is converted into free radicals to commence crosslink reactions and initiate polymerisation to form hydrogels. The photoinitiator generally used in this process has a high absorption at a specific wavelength of light in order to produce radicals [7]. The photopolymerisation technique has been investigated for various biomedical applications, including drug delivery [8,9], tissue engineering [10], and biosensing [11]. Other applications, including coatings, adhesives, and sealants, have also been reported [12]. Smart material and smart hydrogel research studies are being accelerated exponentially. Smart polymer hydrogels can be produced by photopolymerisation, leading to a change in their structure and volume phase transition, in the presence of external stimuli, resulting in enormous potential for scientific observations and for various advanced technological applications [13].

Smart polymers respond reversibly to stimuli. The application of heat in aqueous environments often results in the solubility of hydrophilic polymers. However, Poly (N-Vinylcaprolactam) (PNVCL) and Poly (N-isopropyl acrylamide) (PNIPAAm) are water-soluble polymers that precipitate when heated in a solution. PNVCL, a temperature-responsive monomer, is a well-studied monomer due to its biocompatibility and low toxicity.

PNVCL’s lower critical solution temperature (LCST) is in the range of 34–37 °C [14,15,16,17,18,19], which is close to the physiological temperature. The LCST is influenced by various factors, such as the molecular weight, polymer concentration in solution, proportion of other comonomers, and structure or morphology [20]. As a result, when PNVCL is injected into the body, it can transform from a liquid to a gel state, thus forming an in situ hydrogel [21]. In comparison to PNVCL, the most popular temperature-responsive monomer is Poly (N-isopropyl acrylamide) (PNIPAAm) [22]. PNIPAAm is a water-soluble, nonionising polymer that possesses an LCST near body temperature, like PNVCL. However, PNVCL is considered more favourable over PNIPAAm as PNIPAAm produces small amide compounds once hydrolysed, which makes PNIPAAm unfavourable for biomedical applications [23]. PNVCL has demonstrated the ability to inhibit certain strains of pathogenic bacteria, such as *Staphylococcus aureus* (gram-positive), *Escherichia coli* (gram-negative), and the yeast *Candida albicans*. Therefore, PNVCL is of great interest within biomaterials and tissue engineering [20].

This study proposes to examine the effect of diethylene glycol diacrylate (DEGDA) on the temperature-responsive monomer, PNVCL. DEGDA was selected as a crosslinking agent, and Vinylacetate (VAc) and N-Vinylpyrrolidone (NVP) were incorporated as the comonomers. The resulting blends—PNVCL, PNVCL-DEGDA, and PNVCL-DEGDA-VAc/NVP-based hydrogels—were prepared by using photopolymerisation in the presence of a Diphenyl (2,4,6-trimethyl benzoyl) phosphine oxide (TPO) photoinitiator. FTIR analysis identifies the functional groups within the NVCL-based copolymers and determines the success of photopolymerisation. DSC analysis shows that the increased DEGDA crosslinker within the NVCL-based hydrogels can lead to a decrease in the glass transition temperature (T_g_) of the hydrogels. Swelling analysis displays that the lower the concentration of crosslinker present in the hydrogel, the quicker the hydrogels reach their maximum swelling ratio.

## 2. Results and Discussion

### 2.1. Photopolymerisation of PNVCL Hydrogels

Photopolymerisation is a method of polymerisation where visible or UV light is used to form in situ crosslinkages in order to polymerise a sample [24]. Compared to other polymerisation techniques, photopolymerisation has many advantages such as producing minimal heat during the process and being a relatively fast process, with polymerisation occurring within seconds to several minutes [25].

**Figure 1:**
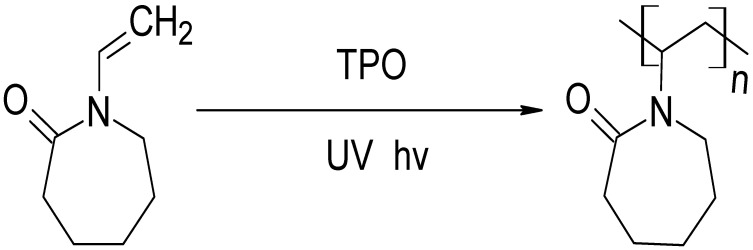
Schematic representation illustrating the process of photopolymerisation, showing the conversion of NVCL into PNVCL in the presence of TPO photoinitiator.

The homopolymer was photopolymerised in the presence of 1 wt% TPO, and the copolymers—PNVCL/DEGDA, PNVCL/DEGDA/VAc, and PNVCL/DEGDA/NVP—were photopolymerised in the presence of a 0.1 wt% TPO photoinitiator. TPO initiating the polymerisation of the homopolymer at 0.1 wt% was too small of an amount to initiate polymerisation. A minimal amount of photoinitiator, 0.1 wt%, was used for the additional samples in an attempt to limit the cytotoxicity of samples. TPO can decompose, thereby generating free radicals, when exposed to UV light. The polymerisation process of the monomers is initiated by the free radicals, which are generated by the TPO photoinitiator. Figure 1 depicts the reaction mechanism for polymerising the NVCL monomer in the presence of the TPO photoinitiator.

**Figure 2:**
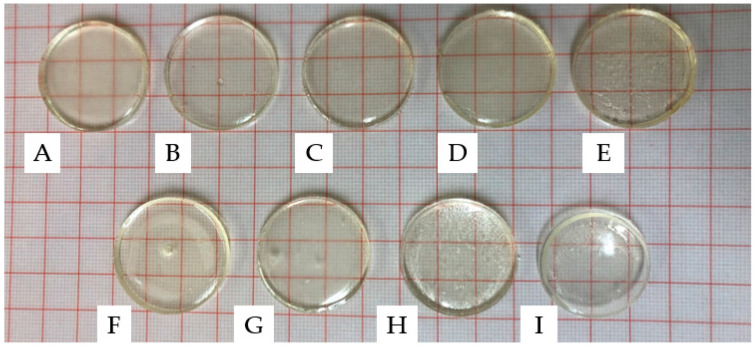
PNVCL xerogels produced using different ratios of DEGDA/VAc/NVP with 0.1 wt% TPO: (**A**) S1, (**B**) S2, (**C**) S3, (**D**) S4, (**E**) S5, (**F**) S6, (**G**) S7, (**H**) S8, and (**I**) S9.

To prepare the samples, formulations, as listed in Section 4.2, were used and poured into a silicone mould with disc-shaped cavities. Prior to removal from the mould, the samples were dried for 24 h in a vacuum oven. The circular discs had a thickness of 4 mm and a diameter of 30 mm. As can be observed in Figure 2, all samples were well cured and maintained their structural integrity after removal from the mould. The produced discs had a glass-like appearance with a high level of transparency. In semi-crystalline polymers, the presence of crystalline regions can lead to light scattering, resulting in opacity. The degree of crystallinity, distribution, and size of crystalline regions can also impact the level of opacity [26]. The transparency of the produced disc samples suggests that they were amorphous, with polymer chains randomly distributed and lacking distinct boundaries [27,28].

### 2.2. Attenuated Total Reflectance–Fourier Transform Infrared Spectroscopy

**Figure 3:**
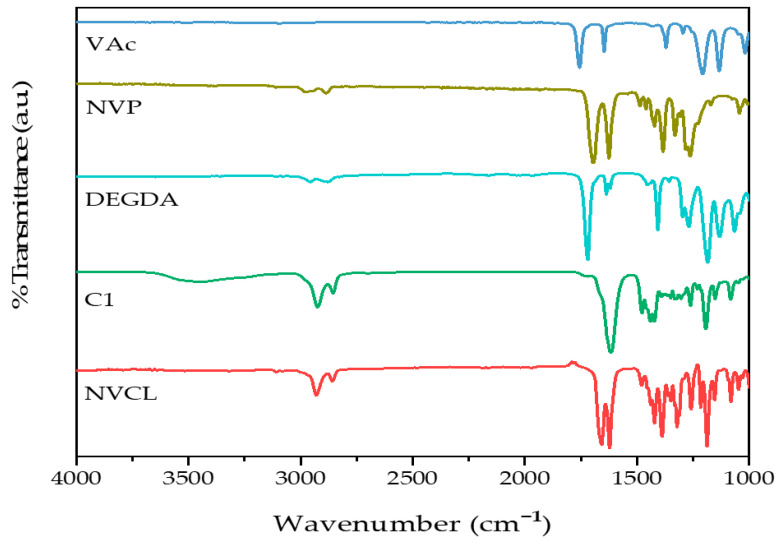
FTIR spectra of (top–bottom) VAc monomer, NVP monomer, DEGDA monomer, PNVCL100, and NVCL neat.

**Figure 4:**
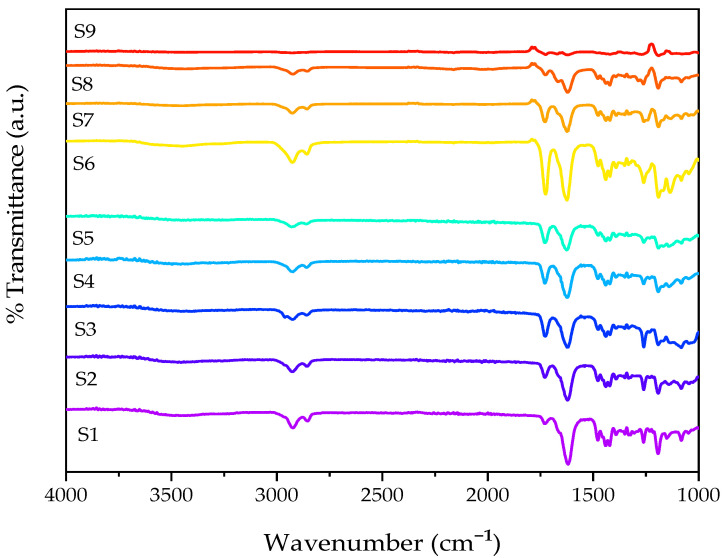
FTIR spectra of (top–bottom) S9, S8, S7, S6, S5, S4, S3, S2, and S1.

To examine the differences in chemical structures between monomers and photopolymerised samples, ATR-FTIR was utilised. The absorbed radiation is unique to the functional groups and chemical bonds in the sample and can be used to identify them [29]. In this study, ATR-FTIR was employed to differentiate between the chemical bonds present in the monomers (NVCL, DEGDA, VAc, and NVP) and the polymerised samples (S1, S2, S3, S4, S5, S6, S7, S8, and S9). The results of the IR spectra and the assignment of absorption bands to functional groups are presented in Figure 3 and Figure 4 and Table 1, respectively.

The NVCL monomer displayed characteristic carbonyl peaks at 1659 cm^−1^ and 1625 cm^−1^. These absorption bands were assigned to the C=O functional group and C=C for the NVCL monomer [30]. C–N stretching vibrations at 1255 cm^−1^ and 1045 cm^−1^ and C–H stretching vibrations at 2927 cm^−1^ and 2855 cm^−1^ were observed for the NVCL monomer. Absorption bands were observed at 1618 cm^−1^, 1196 cm^−1^, and 2919 cm^−1^ for the homopolymer PNVCL; these bands were assigned as the stretching vibrations of the C=O group in the amide functional group, the C–O–C group in the ester functional group (–CO–O–), and the C–H functional group, respectively. The amide^−1^ band, located at 1618 cm^−1^, is a characteristic absorption band of all amides, and its precise position is influenced by various factors, such as the physical state of the compound and the extent of hydrogen bonding present in the sample [17,31].

The characteristic absorption bands at 1648 cm^−1^ and 1740 cm^−1^ (VAc) and 1624 cm^−1^ and 1383 cm^−1^ (NVP) were observed for the comonomers. These absorption bands were attributed to the stretching vibration of the acetate group’s (COO–) C=O and the stretching vibration of the carbonyl group in unconjugated ketones for the VAc monomer [32]. The absorption band at 1624 cm^−1^ in NVP was assigned to the C=O stretching vibration of the pyrrolidone group. The NVP monomer displayed an absorption band, which appeared at 1383 cm^−1^, and was assigned to the C–N stretching vibration [33]. Finally, for the DEGDA crosslinker, the assignment at 1720 cm^−1^ was for C=O stretching.

The FTIR spectra of the polymerised PNVCL/DEGDA, PNVCL/DEGDA/VAc, and PNVCL/DEGDA/NVP samples are displayed in Figure 4. PNVCL/DEGDA showed a new peak post-polymerisation at 1618 cm^−1^, which is not characteristic of the homopolymer, thus indicating that copolymerisation had successfully occurred [34]. The stretching vibration of the C=O functional group is observed which can be associated with the formation of new crosslinks between PNVCL and DEGDA. It can be seen that as the proportion of DEGDA in the formulation increases (from 5% to 40%), so too does the intensity of the peak. Post-polymerisation, the distinctive peak assigned to the DEGDA crosslinker at 1720 cm^−1^ disappears, further indicating successful polymerisation. PNVCL/DEGDA/VAc displayed a new peak post-polymerisation at 2853 cm^−1^, which is not characteristic of the monomer. This band was assigned as the C–H stretching functional group, and it can also be seen in S6 and S7 samples. Similar results were obtained in the spectra for PNVCL/DEGDA/NVP. In the spectrum, the polymerised samples containing DEGDA and NVP displayed a new peak at 1725 cm^−1^, which is not characteristic of the monomer, which was subsequently assigned as the stretching vibration of the C=O group in the amide functional group [35].

### 2.3. Differential Scanning Calorimetry

Differential scanning calorimetry (DSC) is a technique in thermal analysis used to examine a material’s response to changes in temperature, heat capacity, and thermal transitions. In this study, DSC analysis was carried out to investigate the impact of DEGDA crosslinker and comonomers (VAc/NVP) on the thermal characteristics of PNVCL hydrogels.

**Figure 5:**
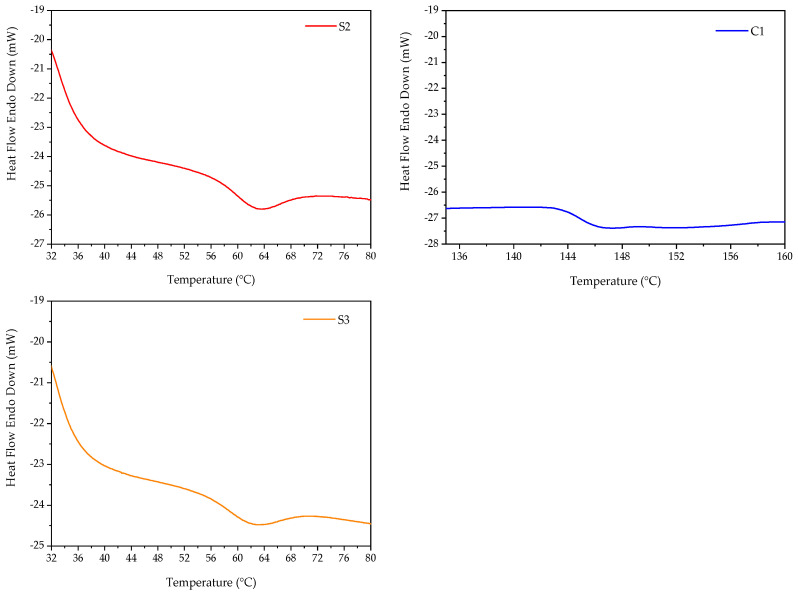
A representative thermograph of samples C1, S2, and S3.

Figure 5 displays the thermograph of sample C1, P(NVCL100), which exhibited a T_g_ of 146 °C. PNVCL has a T_g_ of 147 °C and is considered an amorphous polymer [25,26,27]. Several factors, including the molecular weight, purity, presence of moisture, and disparity, have been reported to affect the T_g_ of PNVCL [36,37,38]. The T_g_ of copolymers will commonly fall between those of the homopolymers as the copolymers have intermediate chain stiffness and interchain attraction [39]. The incorporation of a copolymer can disrupt the molecular packing, leading to a reduction in interchain forces of attraction and increased disorder. As a result, the predicted T_g_ of copolymers may be higher than the actual T_g_ observed [40].

The T_g_ is a crucial factor to consider as it affects the diffusion rates of a polymer [41]. Below the T_g_, the diffusion rates of polymers are decreased, while above T_g_, they are increased. Above the T_g_, the polymer undergoes dissolution, leading to the formation of a gel layer at the dissolving interface. At this interface, polymer chains become disentangled and diffuse into the surrounding medium. On the other hand, at temperatures below T_g_, the thickness of the gel layer reduces, leading to a change in the dissolution mechanism from chain disentanglement to an eruption process where small blocks of polymer are released [42].

The DSC of S2 and S3, as displayed in Figure 5, featured a T_g_ value of 65.80 °C and 64.54 °C, respectively. The T_g_ value of the NVCL-based copolymers was found to be influenced by the amount of DEGDA crosslinker present in the copolymer. As the concentration of the DEGDA crosslinker increased, the T_g_ value decreased.

### 2.4. Pulsatile Swelling Studies

The swelling and solubility properties of the physically and chemically crosslinked xerogels were investigated under different environmental conditions to examine the effect of variable temperature. These variable conditions were ambient room temperature (20 °C) and elevated temperature (50 °C). Chemically crosslinked xerogels consist of permanent networks when formed. In aqueous media, the polymers exhibit a volume phase transition, where they undergo swelling and shrinking within a critical temperature range. In comparison, physically crosslinked xerogels swell upon contact with a thermodynamically compatible solvent, dissolving over time [43]. Swelling analysis was conducted on PNVCL, PNVCL/DEGDA, PNVCL/DEGDA/VAc, and PNVCL/DEGDA/NVP hydrogels. The homopolymer, C1, physically crosslinked hydrogel lost structural integrity after 3 h and was completely solubilised within 6 h. Below the LCST, the dissolution of the polymer can be attributed to the hydrogen bonding between the polymer and water molecules [44]. Physically crosslinked polymers tend to increase their water solubility as the temperature increases. Polymers with LCST undergo the opposite behaviour; thereby, as the temperature increases, the water solubility decreases. This phenomenon can be explained by the weakening of hydrogen bonds and the prevalence of hydrophobic groups, resulting in polymer precipitation [45].

**Figure 6:**
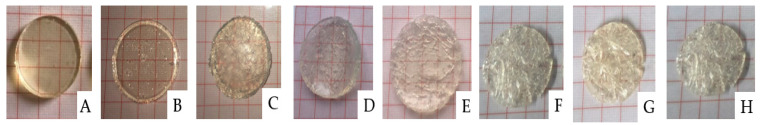
The appearance of the hydrogel swelling for sample S1 over 262 h at times (**A**) 0 h, (**B**) 2 h, (**C** 29 h, (**D**) 48 h, (**E**) 75 h, (**F**) 100 h, (**G**) 139 h, and (**H**) 262 h. (**A**–**E**) represent the hydrogels at room temperature, and (**F**–**H**) represent the hydrogels at 50 °C.

**Figure 7:**
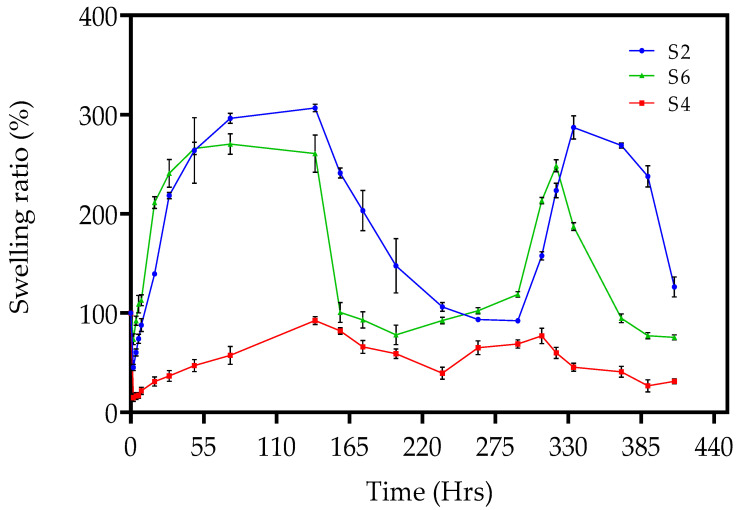
The swelling ratios of chemically crosslinked samples of PNVCL, PNVCL/DEGDA, PNVCL-DEGDA/VAc, and PNVCL-DEGDA/NVP conducted at temperatures below the LCST (20 °C) and above the LCST (50 °C). (●) S2, (■) S4, and (▲) S6.

The chemically crosslinked xerogels—S2, S2, S3, S4, S5, S6, S7, S8, and S9—were investigated under the same conditions. The crosslinking degree is the main factor affecting the swelling behaviour of chemically crosslinked hydrogels [46]. In this study, five different crosslinker concentrations were employed in the fabrication of the chemically crosslinked hydrogels: 5 wt%, 10 wt%, 20 wt%, 30 wt%, and 40 wt%. Previous work by Choong et al. (2017) employed variable content of the DEGDA crosslinker with a range of 10–50 wt% for the fabrication of a high-performance shape memory polymer for SLA 4-D printing [47]. The authors discovered that a concentration of 10 wt% crosslinkers exhibited the best shape memory performance, attaining 100% full recovery and the stability of the shape memory properties. In this study, varying the concentration of crosslinker was conducted to achieve the optimum degree of swelling for a targeted application. The chemically crosslinked hydrogels were initially transparent at 20 °C. However, over time and upon immersion in distilled water at 50 °C, the hydrogels changed from transparent to opaque. Figure 6 illustrates the visual change in the hydrogel, sample S1, over 262 h at room temperature and 50 °C. Holback et al. (2011) reported similar findings and attributed them to the hydrogel’s surface being more compact than the interior of the gel matrix [48]. Figure 7 displays the swelling ratio of S2, S4, and S6, at temperatures below the LCST of (20 °C) and above the LCST of (50 °C) until three cycles were completed. It was observed that the lower the concentration of crosslinker present in the hydrogel, the quicker the hydrogels reached their maximum swelling ratio [29]. For example, sample S2, consisting of 10 wt% crosslinker concentration, had reached the maximum swelling ratio after 29 h. In comparison, S4, with a crosslinker concentration of 30 wt%, did not reach the maximum swelling ratio after 75 h. Figure 7 also illustrates the pulsatile swelling of sample S6; by maintaining a low crosslinker density (10 wt%) and by incorporating a comonomer, sample S6 maintained structural integrity over the three cycles. The literature has reported that the higher crosslink density increases resistance to chain extension in the hydrogel structure, which, in turn, reduces the equilibrium swelling degree. A hydrogel with a lower crosslink density exhibits a more porous structure, which promotes faster swelling and deswelling rates by facilitating the diffusion of water into and out of the hydrogel matrix [43].

### 2.5. Gel Fraction Measurement

**Figure 8:**
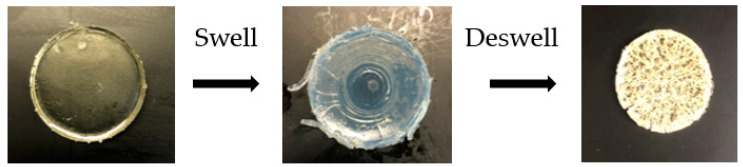
The process of determining gel fraction percentage of S2.

**Figure 9:**
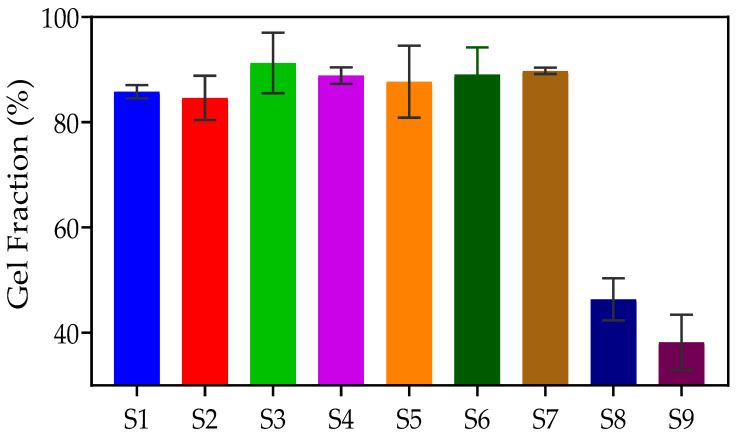
Comparison of the Gel fraction among S1, S2, S3, S4, S5, S6, S7, S8, and S9.

The gel fraction is a useful qualitative measure of the effectiveness of network formation in hydrogels. The gel fraction indicates the number of crosslinks that occur between the polymers in the hydrogel. A higher gel fraction percentage is indicative of a greater degree of covalent bonding. The higher density of formed crosslinks additionally leads to a reduction in the swelling capability of the hydrogel, i.e., the ability of water to enter/exit the hydrogel structure is reduced [49]. Figure 8 illustrates the process of determining the gel fraction percentage of S2. The percentages of gel fraction for PNVCL/DEGDA-, PNVCL/DEGDA/Vac-, and PNVCL/DEGDA/NVP-based hydrogels are displayed in Table 2 and Figure 9. The results indicate that the PNVCL/DEGDA-based samples maintained a gel fraction in the range of 85% to 91%. Similarly, the PNVCL/DEGDA/VAc samples showed a high gel fraction percentage. The P(NVCL70-DEGDA10-VAc20) (S6) sample maintained a gel fraction percentage of 89.08. By increasing the concentration of VAc to 40 wt%, S7 maintained a gel fraction of 89.76%. However, the gel fraction percentage was much lower for the PNVCL/DEGDA/NVP-based samples. S8 displayed a gel fraction measurement of 46.36%. Again, by increasing the concentration of NVP to 40 wt%, the gel fraction measurement of S9 decreased to 38.18%.

### 2.6. Goniometry

The hydrophobicity level of each hydrogel was determined by measuring its contact angle. To evaluate the wettability of the hydrogel surface, goniometry was used to measure the contact angle between a water droplet and the dry surface of the hydrogel. Samples with a contact angle closer to 0° were considered more hydrophilic, while those with a contact angle up to 90° were classified as hydrophilic. Conversely, samples with a contact angle greater than 90° were considered hydrophobic, and surfaces with contact angles above 150° were regarded as superhydrophobic, indicating that the liquid droplet meets the surface without significant wetting [27].

**Figure 10:**
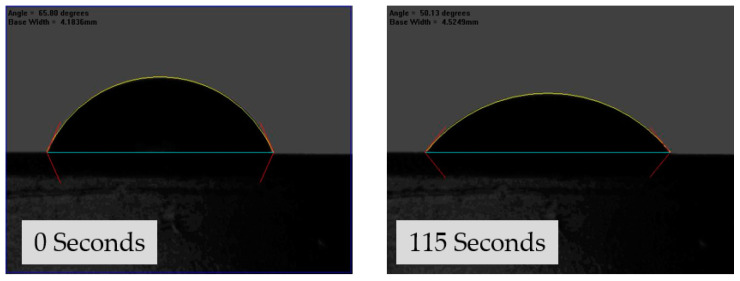
The contact angle of sample S2 after 0 and 115 s.

**Figure 11:**
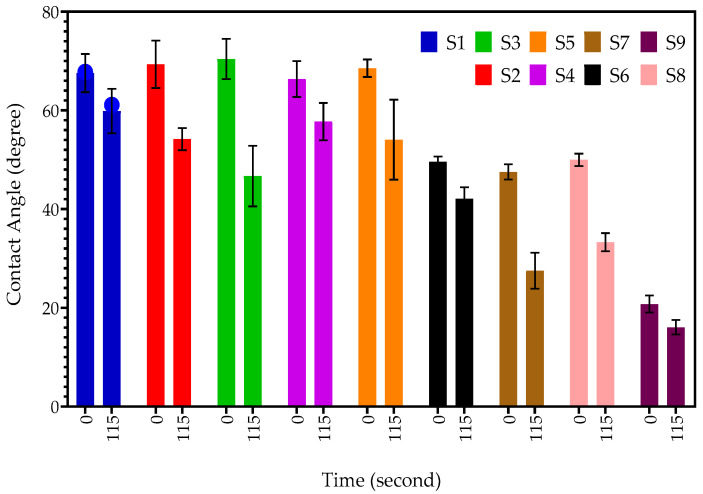
The contact angles of samples S1, S2, S3, S4, S5, S6, S7, S8, and S9 from 0 to 115 s.

Figure 10 demonstrates the contact angle of sample S2 after 0 s and 115 s. The contact angle at 0 s for S2 was 65.80°, which decreased to 50.13° after 115 s. Sample S1, which exhibited a mean contact angle of 67.5° at 0 s, transitioned into a droplet with a mean contact angle of 59.86°, suggesting that the hydrogel is hydrophilic. Similarly, increasing the concentration of the DEGDA crosslinker in sample S5 resulted in a mean contact angle of 68.53° at 0 s, which decreased to a mean contact angle of 54.07° at 115 s, indicating that the hydrogel became more hydrophilic. When the concentration of crosslinker in the hydrogel is increased, there is no notable change in the contact angle at 0 seconds from S1 to S5. However, a decrease in the contact angle is observed at 115 seconds for S1, S2, and S3, which slightly increases with S4 and S5. Sample S6, S7, S8, and S9 incorporated with either VAc or NVP showed a decrease in the mean contact angle in comparison to samples incorporated with only a DEGDA crosslinker. Sample S6, consisting of VAc of 20 wt%, displayed a mean contact angle of 49.59° at 0 s, which decreased to 42.10° at 115 s. By increasing the concentration of VAc to 40 wt%, sample S7, the mean contact at 0 s was determined to be 47.53°, which rapidly decreased to 27.51°, indicating that the S7 hydrogel was more hydrophilic than sample S6. Likewise, it was determined that the copolymers incorporated with NVP, sample S8, and sample S9 were the most hydrophilic tested samples. Figure 11 displays the contact angle of the samples at 0 s and 115 s.

### 2.7. Tensile Testing

**Figure 12:**
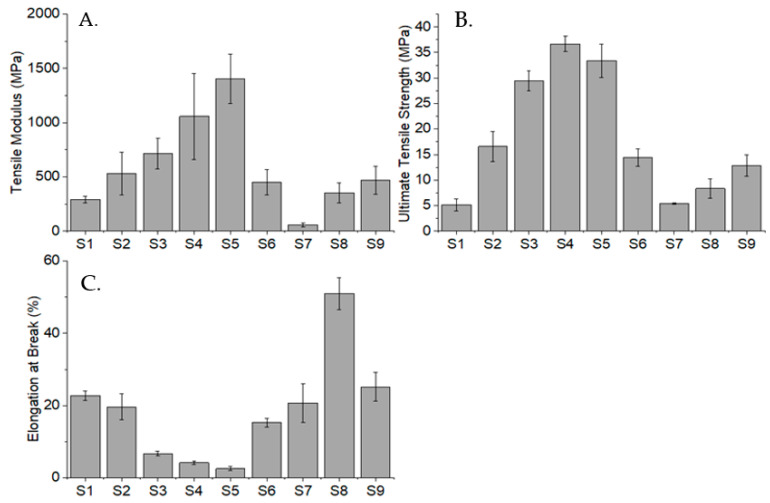
Tensile property comparison between samples S1, S2, S3, S4, S5, S6, S7, S8, and S9. (**A**) Tensile modulus, (**B**) Ultimate tensile strength, (**C**) Elongation at break.

The tensile test was performed on samples S1, S2, S3, S4, S5, S6, S7, S8, and S9 to investigate the effect of incorporating different comonomers with NVCL on their tensile modulus, ultimate tensile strength (UTS), and percentage elongation, at break. The results of the tensile test are illustrated in Figure 12. It is worth noting that the deviation in the results may be on account of the photopolymerisation process that is distinct to an injection moulding process, which provides holding pressure during the fabrication of samples. The absence of holding pressure could cause the fabricated copolymers to have porosity and a weaker overall polymer structure [50].

The result shows an increasing trend in the DEGDA-containing copolymers. As the DEGDA concentration increases, the tensile modulus increases too. The same pattern can be observed in their UTS; however, there is a slight decrease in S5 samples that contained 40 wt% of DEGDA. This could be due to the excessive DEGDA concentration that led to poorer polymer chain arrangement of the copolymer. Conversely, the concentration of DEGDA shows a reverse effect in the sample’s percentage elongation. This proves that through the incorporation of DEGDA, the samples became stiffer and less stretchable. Based on the literature, the copolymer consisting of 90 wt% NVCL and 10 wt% VAc exhibits a modulus of around 1 GPa, a UTS of 29 MPa, and a percentage elongation of 2.6% [51]. Correspondingly, the fabricated S6 and S7 samples that contained 20% and 40% of VAc show a decreasing trend in the tensile modulus and UTS with increasing VAc concentration; yet, such numbers are relatively lower than the literature due to higher VAc concentrations. In fact, VAc has a plasticising effect on the copolymer, which is further proven by the increased percentage elongation, apart from the weakened tensile modulus and UTS, when its concentration is increased. Finally, the incorporation of the NVP monomer has an increasing effect on the tensile modulus and UTS but a decreasing effect on the percentage elongation of the copolymer. The improved copolymer stiffness may be owing to the better polymer conformation resulting from the incorporation of the comonomer. In conclusion, tensile properties are greatly altered by different types and concentrations of comonomers.

## 3. Conclusions

This study aimed to enhance the understanding of the synthesis and characterisation of NVCL-based copolymers chemically crosslinked with DEGDA by using UV photopolymerisation. The hydrogels were characterised by employing the following methods: DSC, pulsatile swelling studies, tensile testing, goniometry, and gel fraction measurement. The findings reveal that the T_g_ of the copolymers was influenced by the concentration of crosslinker, with higher levels of DEGDA leading to a decrease in the T_g_. The tensile testing indicates that the tensile properties were greatly altered by different types and concentrations of comonomers; it was found that as the DEGDA concentration increased, the tensile modulus increased. The pulsatile swelling studies identified S2 as the most promising copolymer for investigating the 3-D printability of these copolymers. Ongoing research is being conducted, and the findings will be presented at a later time.

## 4. Materials and Methods

### 4.1. Materials

The main material used in this study is N-vinylcaprolactam (NVCL), which has a molecular weight of 139.19 g/mol, and was provided by Sigma Aldrich, Co. Dublin, Ireland, and should be stored at a temperature of 2 to 8 °C. The photoinitiator Diphenyl (2,4,6-trimethylbenzoyl) phosphine oxide (TPO) was also obtained from Sigma Aldrich, Co. Dublin, Ireland. Diethylene glycol diacrylate (DEGDA) with a molecular weight of 214.22 g/mol, obtained from Sigma Aldrich, Co. Dublin, Ireland, was used as the crosslinker. VAc and NVP, with molecular weights of 86.09 g/mol and 111.14 g/mol, respectively, were also obtained from Sigma Aldrich, Co. Dublin, Ireland. The chemical structures of these materials are listed below in Table 3.

### 4.2. Synthesis of Thermosensitive PNVCL Hydrogel

A Dr. Gröbel UV-Elektronik GmbH UV curing system, Ettlingen, Germany, was used to photopolymerise all samples. This system has an irradiation chamber with 20 UV tubes that emit light with a wavelength between 315 and 400 nm at an average intensity of 10–13.5 mW/cm^2^. Prepolymerised mixtures of specific amounts of DEGDA, VAc, NVP, and NVCL were prepared, and TPO photoinitiator was added at a concentration of 0.1 wt% to ensure sample consistency. The mixtures were then stirred using a magnetic stirrer for 20 min and transferred to a disc-like silicone mould, which was placed horizontally under the UV tubes. The solutions were then cured for 30 min, during which the samples were turned to ensure uniform exposure to light radiation. After complete curing, the samples were subjected to drying in a vacuum oven at 50 °C for 24 h before being utilised. Table 4 illustrates the sample compositions for all PNVCL-based samples produced.

### 4.3. Attenuated Total Reflectance–Fourier Transform Infrared Spectroscopy

A Perkin Elmer Spectrum One FT-IR Spectrometer (C-001), Waltham, MA, USA, equipped with a universal ATR sampling accessory was used to perform Attenuated Total Reflectance–Fourier Transform Infrared Spectroscopy (ATR-FTIR) analysis. The analysis was carried out at a temperature of approximately 22 °C, covering a spectral range of 4000–650 cm^−1^. A fixed universal compression load of 75 N was applied with 4 scans performed per sample cycle.

### 4.4. Differential Scanning Calorimetry

Differential scanning calorimetry (DSC) was used for the purpose of observing the thermal transitions of the materials. A Sartorius balance (Sartorius, Goettingen, Germany) with a resolution of 0.01 mg was used to weigh an appropriate amount of sample, which ranged from 8 to 12 mg, and was placed in hermetically sealed aluminium pans. The pans were crimped before testing. A TA Instruments DSC 2920, New Castle, DE, USA, modulated differential scanning calorimeter was used for the analysis. Prior to the analysis, the instrument was calibrated using indium as a standard, and scans were performed at a rate of 10 °C/min, ranging from 20 to 200 °C. To remove volatiles from the purging head, nitrogen gas was used at a flow rate of 30 mL/min.

### 4.5. Tensile Testing

Tensile testing is a destructive test process that provides materials physical information such as the ultimate tensile strength (UTS), strain properties, and Young’s modulus. ASTM standard D 638 Type V tensile bars were premade via UV curing, utilising a silicone mould. Uniaxial tensile testing was performed using a Zwick Roell (Ulm, Germany) Z010 tensile testing machine equipped with a 10 kN load cell and an optical extensometer. In line with ASTM standard D 638 for Type V tensile bars, the grips gap was set to 25.4 mm, and the specimens were tested at a rate of 1 mm/min. Each sample was measured in five copies until failure occurred.

### 4.6. Pulsatile Swelling Studies

After photopolymerisation, the chemically crosslinked samples were subjected to a 24 h vacuum drying process at 50 °C. The dry weight of the polymerised samples was determined initially using a Sartorius balance with a resolution of 1 × 10^−5^ and represented as W_d_. After preparation, the samples were placed into glass Petri dishes that contained 25 mL of distilled water with a pH of 7.1. The samples were then tested at both room temperature and 50 °C. At specific time intervals, the samples were removed from the Petri dishes, and any excess surface water was removed using filter paper. The remaining weight, recorded as “W_t_”, represented the wet weight of the samples. All samples were tested in triplicate. The swelling ratio was determined through the utilisation of Equation (1):

Swelling Ratio (%) = ((W_t_ − W_d_)/W_d_) × 100 (1)

where W_t_ refers to the weight of the gel at a specified time, while W_d_ represents the mass weight of the polymer in its dry state.

### 4.7. Gel Fraction Measurement

The efficiency of hydrogel network formation can be quantitatively evaluated using the gel fraction measurement [23]. To measure the gel fraction of all batches, a round disc was used. The xerogel samples were placed in covered Petri dishes containing 30 mL of distilled water and left at room temperature until they reached a state of equilibrium swelling. Once equilibrium swelling was achieved, the samples were dried in a vacuum oven at 50 °C and 100 Pa until no further changes in weight were observed. The gel fraction percentage of the samples was then calculated using the following formula:

Gel fraction (%) = (W_d_/W_0_) × 100 (2)

where W_0_ refers to the initial weight of the dried sample, while W_d_ represents the weight of the dried insoluble portion of the sample after it has been extracted with water.

### 4.8. Goniometry

The ability of a solid substrate to resist liquids was evaluated using contact angle goniometry [52]. A dynamic sessile droplet of water was applied to the surface of the xerogels, while photographs were taken to record the spread of the droplet. The samples were placed on a stage for the experiment [26]. The angle measurement was obtained from the photo screen automatically. To assess the wettability of the xerogels, photos taken at 0 s and 115 s were analysed in this study. All experiments were performed in triplicate.

### 4.9. Statistical Analyses

GraphPad Prism version 8.0.1. for Windows, developed by GraphPad Software in San Diego, CA, USA, was used for data handling and analysis. The software was used to input all test data, and the mean and standard deviations were calculated for the replicate data sets. All data presented in the figures are shown as the mean value with error bars indicating standard deviation, unless otherwise specified.

## Figures and Tables

**Table 1:** Identification and interpretation of characteristic peaks obtained from ATR-FTIR analysis.

	Sample	Wavelength (cm^−1^)	Functional Group
**Monomers**	NVCL	1659	C=O
		1625	C=C
		2927, 2855	C–H
	DEGDA	1720	C=O
	VAc	1648	C=O
		1740	C–H
	NVP	1624	C=O
		1383	C–N–C
**Polymerised Samples**	PNVCL	1618	C=O
		1196	–CO–O-
		2919	C–H
	PNVCL/DEGDA	1618	C=C
	PNVCL/DEGDA/VAc	2853	C–H
	PNVCL/DEGDA/NVP	1725	C=O

**Table 2:** Gel Fraction % for PNVCL/DEGDA-, PNVCL/DEGDA/Vac-, and PNVCL/DEGDA/NVP-based samples.

ID Code	Gel Fraction (%)
S1	85
S2	84
S3	91
S4	88
S5	87
S6	89
S7	89
S8	46
S9	38

**Table 3:** Name and chemical structure of materials selected.

Material	Chemical Structures
N-vinylcaprolatam (NVCL)	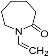
Diphenyl(2,4,6-trimethylbenzoyl) phosphine oxide (TPO)	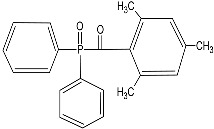
Di(ethylene glycol) diacrylate (DEGDA)	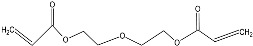
Vinylacetate (VAc)	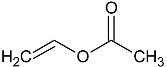
N-vinylpyrrolidone (NVP)	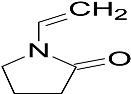

**Table 4:** Code and compositions of copolymerised samples subsequent to photopolymerisation.

ID CODE	Formulation	Photoinitiator	Monomer	Crosslinker	Comonomers	
		TPO (wt%)	NVCL (wt%)	DEGDA (wt%)	VAc (wt%)	NVP (wt%)
C1	P(NVCL100)	1	100	--	--	--
S1	P(NVCL95-DEGDA5)	0.1	95	5	--	--
S2	P(NVCL90-DEGDA10)	0.1	90	10	--	--
S3	P(NVCL80-DEGDA20)	0.1	80	20	--	--
S4	P(NVCL70-DEGDA30)	0.1	70	30	--	--
S5	P(NVCL60-DEGDA40)	0.1	60	40	--	--
S6	P(NVCL70-DEGDA10-VAc20)	0.1	70	10	20	--
S7	P(NVCL50-DEGDA10-VAc40)	0.1	50	10	40	--
S8	P(NVCL70-DEGDA10-NVP20)	0.1	70	10	--	20
S9	P(NVCL50-DEGDA10-NVP40)	0.1	50	10	--	40
